# Neutralizing Antibodies against Enteroviruses in Patients with Hand, Foot and Mouth Disease 

**DOI:** 10.3201/eid2602.190721

**Published:** 2020-02

**Authors:** Lam Anh Nguyet, Tran Tan Thanh, Le Nguyen Thanh Nhan, Nguyen Thi Thu Hong, Le Nguyen Truc Nhu, Hoang Minh Tu Van, Nguyen Thi Han Ny, Nguyen To Anh, Do Duong Kim Han, Ha Manh Tuan, Vu Quang Huy, Ho Lu Viet, Hoang Quoc Cuong, Nguyen Thi Thanh Thao, Do Chau Viet, Truong Huu Khanh, Louise Thwaites, Hannah Clapham, Nguyen Thanh Hung, Nguyen Van Vinh Chau, Guy Thwaites, Do Quang Ha, H. Rogier van Doorn, Le Van Tan

**Affiliations:** Oxford University Clinical Research Unit, Ho Chi Minh City, Vietnam (L.A. Nguyet, T.T. Thanh, N.T.T. Hong, L.N.T. Nhu, H.M.T. Van, N.T.H. Ny, N.T. Anh, D.D.K. Han, L. Thwaites, H. Clapham, G. Thwaites, D.Q. Ha, H.R. van Doorn, L.V. Tan);; Children’s Hospital 1, Ho Chi Minh City (L.N.T. Nhan, T.H. Khanh, N.T. Hung);; Children’s Hospital 2, Ho Chi Minh City (H.M. Tuan, H.L. Viet, D.C. Viet);; University of Medicine and Pharmacy, Ho Chi Minh City (H.M. Tuan, V.Q. Huy);; Pasteur Institute, Ho Chi Minh City (H.Q. Cuong, N.T.T. Thao);; University of Oxford, Oxford, UK (L. Thwaites, H. Clapham, G. Thwaites, H.R. van Doorn);; Hospital for Tropical Diseases, Ho Chi Minh City (N.V.V. Chau)

**Keywords:** hand foot and mouth disease, enterovirus, enterovirus A71, neutralization, coxsackievirus, viruses, Vietnam, HFMD

## Abstract

Hand, foot and mouth disease (HFMD) is an emerging infection with pandemic potential. Knowledge of neutralizing antibody responses among its pathogens is essential to inform vaccine development and epidemiologic research. We used 120 paired-plasma samples collected at enrollment and >7 days after the onset of illness from HFMD patients infected with enterovirus A71 (EV-A71), coxsackievirus A (CVA) 6, CVA10, and CVA16 to study cross neutralization. For homotypic viruses, seropositivity increased from <60% at enrollment to 97%–100% at follow-up, corresponding to seroconversion rates of 57%–93%. Seroconversion for heterotypic viruses was recorded in only 3%–23% of patients. All plasma samples from patients infected with EV-A71 subgenogroup B5 could neutralize the emerging EV-A71 subgenogroup C4. Collectively, our results support previous reports about the potential benefit of EV-A71 vaccine but highlight the necessity of multivalent vaccines to control HFMD.

Since 1997, hand, foot and mouth disease (HFMD) has emerged as a serious childhood infection in the Asia–Pacific region (*1*,*2*) with the potential of spreading to other parts of the world. Indeed, HFMD epidemics, especially those caused by enterovirus A71 (EV-A71), have increasingly been reported worldwide, including in the United States and Europe (*3*–*5*). Although HFMD is a mild infection in most cases, severe clinical complications (e.g., central nervous system involvement as brainstem encephalitis) may happen and can be fatal (*1*,*6*). However, no antiviral drugs are available to the affected patients, including those with severe clinical phenotypes.

HFMD is caused by various serotypes of enterovirus A of the family Picornaviridae. Of these, EV-A71, coxsackievirus A (CVA) 6, CVA10, and CVA16 are the most common pathogens isolated from patients with clinically suspected HFMD, with CVA6 being increasingly reported (*7*–*9*). In Vietnam, our recent report showed that of 1,547 patients with HFMD enrolled in a clinical study, EV-A71 was detected in 24.4%, followed by CVA6 (21.8%), CVA16 (10.8%), and CVA10 (7.9%). Other enteroviruses detected sporadically included CVA4 (1.7%), CVA12 (1.4%), and CVA2 (0.6%) (*10*). Infection with EV-A71 has received more attention because it frequently causes severe HFMD, especially in recent outbreaks recorded in the Asia–Pacific region since 1997 (*6*,*11*). Consequently, inactivated monovalent vaccines for EV-A71 have been successfully developed and licensed in China (*12*–*14*). The use of those vaccines, however, has been voluntary and restricted within mainland China.

Because the viruses causing HFMD are diverse, ongoing efforts exist to develop multivalent vaccines, especially those including antigens of the aforementioned common serotypes (*15*). Results from these preclinical studies using animal models showed a lack of cross-reactivity among EV-A71, CVA6, CVA10, and CVA16 (*16*,*17*). There is, however, scarce information about to what extent human infection with 1 HFMD-causing enterovirus serotype can elicit (cross-)neutralizing antibodies against homotypic and heterotypic enterovirus serotypes. Such data are of paramount importance to support the development of intervention strategies (including vaccines) and the design of epidemiologic research on surveillance and transmission dynamics of HFMD and will contribute to the expanded knowledge about host–pathogen and pathogen–pathogen interaction of this emerging clinical problem. We aim to fill the existing gaps in knowledge about seropositivity and (cross-)neutralization elicited as a consequence of human infection by EV-A71, CVA6, CVA10, and CVA16, the 4 most common serotypes responsible for the ongoing epidemic of HFMD worldwide, especially in Asia.

## Materials and Methods

### Settings

The clinical and patient data used in this study were derived from an ongoing clinical study of HFMD that has been conducted at Children’s Hospital (CH) 1, CH2, and the Hospital for Tropical Diseases (HTD) in Ho Chi Minh City, Vietnam, since 2013 (*6*,*8*). These hospitals are tertiary referral centers for children with HFMD in Ho Chi Minh City and southern Vietnam, covering a catchment population of >40 million.

### Patient Enrollment and Data Collection

We screened all patients <12 years of age who came to outpatient departments or were admitted to inpatient wards of CH1, CH2, or HTD with a clinical diagnosis of HFMD and, if outpatients, an illness of <3 days for enrollment in our study. We excluded any patient for whom the attending physician believed another diagnosis was more likely.

We collected information regarding demographics, clinical signs/symptoms, clinical grades, treatments, laboratory tests, length of hospital stay, and outcomes. In addition, for enterovirus serotype determination, we sampled acute throat and rectal swabs at enrollment and collected a plasma sample from each participant at enrollment and 7–14 days after enrollment.

### HFMD Clinical Grade Classification

According to the Vietnamese Ministry of Health, HFMD is clinically divided into 4 major grades. Grade 1 is assigned to patients with mouth ulcers or vesicles/papules on hands, feet, or buttocks, with or without mild fever (<39°C). Grade 2 is further divided into grade 2A (central nervous system [CNS] involvement, myoclonus reported by parents or caregivers only, fever >39°C or ataxia), grade 2B1 (myoclonus observed by medical staff or history of myoclonus and lethargy or pulse >130 bpm), and grade 2B2 (ataxia, cranial nerve palsies, limb weakness, nystagmus, persistent high fever, or pulse >150 bpm). Grade 3 involves autonomic dysfunction with sweating, hypertension, tachycardia, and tachypnea. Grade 4 is for disease with additional cardiopulmonary compromise with pulmonary edema or shock syndrome (*18*). Patients with grade 2B1 or above are considered to have severe HFMD, and often require intravenous immunoglobulin administration.

### Determination of Enterovirus Serotype and EV-A71 Subgenogroup

We determined enterovirus serotype using a combination of PCR and sequencing approaches (*19*–*21*). In brief, we first extracted viral RNA from throat/rectal swab specimens. We then used a 1-step multiplex real-time reverse transcription PCR assay to simultaneously detect all enterovirus serotypes and EV-A71 (*19*). We then tested all specimens positive for enterovirus serotype or EV-A71 to further identify specific enterovirus serotypes or EV-A71 subgenogroups, using a combination of viral protein (VP) 1 PCR and sequencing of the obtained PCR amplicon (*18*,*20*,*21*). Finally, we analyzed the obtained VP1 sequences using a previously described online tool to determine enterovirus serotype or EV-A71 subgenogroup (*22*).

### Selection of Patients and Plasma Sample for Microneutralization Assay

From this study, we selected a convenience sample of 120 patients (30 per serotype CVA6, CVA10, CVA16, and EV-A71) who had plasma samples collected at enrollment and follow-up and were available for immunological response analysis. In addition, for assessment of antigenic difference between subgenogroup B5 (circulating in Vietnam during 2013–2015) and C4 (circulating in Vietnam in 2018), we included 1 available follow-up plasma sample collected from a fatal case, in which the patient was infected with subgenogroup C4 during the 2018 outbreak (*6*).

### Viral Strains

We isolated representative samples of CVA10, CVA16, and EV-A71 (including EV-A71 subgenogroup B5 in 2013 and C4 in 2018) used for microneutralization assay from patients with HFMD who were enrolled in the clinical study (*7*,*8*,*10*). We obtained a CVA6 isolate from the virus archive of Pasteur Institute in Ho Chi Minh City. For EV-A71, unless specified, all neutralization experiments were carried out using EV-A71 subgenogroup B5.

### Microneutralization Assay

We performed the microneutralization as previously described (*23*). In brief, we first inactivated plasma samples at 56°C for 30 min, and we then diluted the samples in serial ratios from 1:8 to 1:512 in maintenance medium (Sigma-Aldrich, https://www.sigmaaldrich.com). Accordingly, the lower limit of detection of the assay was 1:8 and the upper limit was 1:512. Next, we incubated plasma dilutions with an equal volume of 100 times the median culture of infectious dose (TCID_50_) of the virus at 37°C for 1 hour, and then transferred them into a 96-well plate pre-coated with human rhabdomyosarcoma (RD) cells (American Type Culture Collection, https://www.atcc.org). The plate was then incubated in a 5% carbon dioxide incubator at 37°C. Cells were observed daily for cytopathic effects. The antibody titer of the sample was determined by the highest plasma dilution that prevented cytopathic effects in 50% of the wells. We tested each dilution in quadruplicate and included negative and positive controls in each experiment. We defined seropositivity as neutralizing antibody titer >1:8. We defined seroconversion as a change from seronegativity to seropositivity, or at least a 4-fold rise in the neutralizing antibody titer between enrollment and follow-up time points.

### Statistical Analysis

We performed statistical analyses of clinical data using IBM SPSS Statistics 23 (IBM Corp., https://www.ibm.com). We compared categorical variables using a χ^2^ test or Fisher exact test and compared continuous variables using the Mann-Whitney U-test, 1-way analysis of variance (ANOVA) test, or Kruskal-Wallis test. We tested the difference in neutralizing antibody titers between samples obtained at enrollment and follow-up using the Wilcoxon matched-pairs signed rank test, available in Prism 5.04 (GraphPad Software, https://www.graphpad.com).

The institutional review boards of CH1, CH2, and HTD, as well as the Oxford Tropical Research Ethics Committee (OxTREC), approved the study. We obtained written informed consent from a parent or guardian of each enrolled patient.

## Results

### Baseline Characteristics of Patients

We compiled the baseline characteristics and clinical outcome of 120 patients included for analysis of neutralizing antibody responses to all 4 enterovirus serotypes (EV-A71, CVA6, CVA10, and CVA16) (Table 1). All patients were enrolled in the clinical study during July 2013–March 2017. Male patients were predominant (female/male ratio 45/75). Thirteen (10.8%) patients had severe HFMD (grade 2B1 or above); 119 patients (99.2%) made a complete recovery. Following the onset of illness, 50% of the patients were admitted to hospital within 1 day (range 0–5 days) and were enrolled in the clinical study within 2 days (range 0–6 days). All second/follow-up plasma samples included for analysis were collected at day >7 (median 9 days, (range 7–17) after the illness onset.

**Table 1:** Baseline characteristics and clinical outcome of the patients included in study of patients with hand, foot and mouth disease, Vietnam*

Characteristics	All, N = 120	EV-A71, N = 30	CVA6, N = 30	CVA10, N = 30	CVA16, N = 30	p value†
Demographics						
Sex ratio, F/M	45/75	8/22	8/22	14/16	15/15	0.12
Median age, mo (range)	16.2 (1.8–59)	16.7 (4.9–58.6)	16.4 (5.3–59)	14.7 (1.8–41.4)	19 (5.8–46.5)	0.11
Median day of illness from onset (range)						
To hospital admission	1 (0–5)	2 (0–4)	1 (0–3)	1 (0–3)	1 (0–5)	0.058
To enrollment in study	2 (0–6)	2 (0–4)	1.5 (0–3)	2 (0–6)	2 (0–6)	0.802
To collection of second plasma	9 (7–17)	9 (7–12)	9 (7–14)	9 (7–14)	9 (8–17)	0.211
Median day of hospitalization‡	3 (1–12)	4 (2–10)	3 (1–8)	3 (1–6)	4 (2–12)	0.3
Inpatient/outpatient ratio	77/43	17/13	18/12	25/5	17/13	0.08
Clinical characteristics, no. (%)						
Fever	87 (72.5)	24 (80)	17 (56.7)	24 (80)	17 (56.7)	0.16
Cough	26 (21.7)	6 (20)	2 (6.7)	8 (26.7)	10 (33.3)	0.06
Runny nose	21 (17.5)	6 (20)	3 (10)	5 (16.7)	7 (23.3)	0.63
Vomiting	25 (20.8)	7 (23.3)	5 (16.7)	5 (16.7)	8 (26.7)	0.77
Diarrhea	14 (11.7)	3 (10)	3 (10)	3 (10)	5 (16.7)	0.89
Drowsiness	6 (5)	2 (6.7)	0	2 (6.7)	2 (6.7)	0.6
Irritability	14 (11.7)	5 (16.7)	2 (6.7)	5 (16.7)	2 (6.7)	0.46
Myoclonus	30 (25)	10 (33.3)	3 (10)	9 (30)	8 (27)	0.13
Sweating	4 (3.3)	1 (3.3)	1 (3.3)	1 (3.3)	1 (3.3)	1
Lethargy	2 (1.7)	0	0	2 (6.7)	0	0.24
Conjunctivitis	1 (0.8)	0	1 (3.3)	0	0	1
Rash	106 (88.3)	30 (100)	30 (100)	17 (56.7)	19 (63.3)	0
Mouth lesion	111 (92.5)	28 (93.3)	23 (76.7)	30 (100)	30 (100)	0.001
Limb weakness	4 (3.3)	1 (3.3)	1 (3.3)	1 (3.3)	1 (3.3)	0.80
Median pulse, bpm (range)	120 (96–180)	120 (96–180)	120 (100–167)	125 (100–165)	120 (100–170)	0.632
Median blood pressure, mm Hg (range)						
Systolic	90 (75–128)	90 (75–100)	90 (80–120)	90 (80–120)	90 (80–128)	0.661
Diastolic	60 (40–80)	55 (50–62)	55 (40–80)	60 (50–80)	60 (50–77)	0.551
Blood biochemistry results, median (range)						
Leukocyte count, × 10^9^ cells/L	13.1 (1–50.6)	13.6 (7.8–50.6)	12.5 (1–25.9)	14.3 (5–24.7)	12.4 (4.5–24.4)	0.638
Neutrophils, %	51 (2.1–92.7)	51.2 (19.7–72.8)	52.7 (7.7–92.7)	47.6 (2.1–76.3)	50.9 (24.2–86.1)	0.658
Lymphocytes, %	37.1 (3.7–80.2)	37.1 (18.4–65.5)	33.9 (3.7–80.2)	37.6 (17.6–71.2)	36.5 (6.1–60.3)	0.846
Platelet count, × 10^9^/L	322.5 (96–597)	360 (189–522)	341.5 (150–513)	297 (96–452)	293.5 (175–597)	0.086
Glucose, mg/dL	107 (0–212)	112 (62–170)	108.5 (0–154)	108.5 (68–212)	98.5 (51–164)	0.324
C-reactive protein, mg/dL	12.4 (0–102)	4.1 (0–100)	13.7 (0–102)	23.3 (3–58)	9.9 (0–39)	<0.001
Clinical grade, no. (%)						
Mild	107 (89.2)	24 (80)	29 (96.7)	28 (93.3)	26 (86.7)	0.2
Severe	13 (10.8)	6 (20)	1 (3.3)	2 (6.7)	4 (13.3)	
IVIg administration, no. (%)	8 (6.7%)	3 (10)	1 (3.3)	1 (3.3)	3 (10)	0.5
Outcome, no. (%)						
Full recovery	119 (99.2)	30 (100)	29 (96.7)	30 (100)	30 (100)	0.17
Incomplete recovery	1 (0.8)	0	1 (3.3)	0	0	

*CV, coxsackievirus; EV, enterovirus; IVIg, intravenous immunoglobulin. †Results of statistical analyses comparing individual groups of patients infected with EV-A71, CVA6, CVA10, or CVA16. ‡Inpatients only.

Of the 30 patients infected with EV-A71, detailed information about subgenogroup was successfully generated for 13 patients, who were all positive for subgenogroup B5. Of the patients with EV-A71 infections, 20% had severe clinical phenotypes (grade 2B1 or above), whereas severe outcome was recorded in 3.3% of patients infected with CVA6, 6.7% of patients infected with CVA10, and 13.3% of patients infected with CVA16 (Table 1). Mouth lesion distributions and C-reactive protein levels were statistically different among serotypes. Otherwise, there were considerable similarities between groups of patients who were infected with EV-A71, CVA6, CVA10, or CVA16 in terms of clinical characteristics and outcome, as well as blood biochemistry parameters (Table 1), in agreement with a previous report (*8*).

### Seropositivity and Seroconversion

We compiled the results of seropositivity testing at enrollment (baseline) and follow-up (Table 2). Although 60% (18/30) of patients infected with EV-A71 had specific antibodies to EV-A71 at the measured level (>1:16) or above in their blood samples, most of the patients (>70%) infected with CVA6, CVA10, or CVA16 had no specific antibodies to the infecting viruses at enrollment. The proportion of patients with antibodies against heterotypic viruses ranged from 7% (2/30) of CVA6 patients having neutralizing antibodies against EV-A71 to 57% (17/30) of EV-A71 patients having antibodies against CVA10.

**Table 2:** Seropositivity and seroconversion in plasma from hand, foot and mouth disease patients infected with EV-A71, CVA6, CVA10, and CVA16 viruses, Vietnam*

Virus	Immunity status	Virus, no. (%) samples			
		CVA6	CVA10	CVA16	EV-A71
CVA6	Seropositivity				
	At enrollment	2 (7)	10 (33)	5 (17)	2 (7)
	At follow-up	30 (100)	9 (30)	9 (30)	3 (10)
	p value	<0.001	1.0	0.36	1.0
	Seroconversion	28 (93)	1 (3)	7 (23)	2 (7)
CVA10	Seropositivity				
	At enrollment	4 (13)	9 (30)	3 (10)	3 (10)
	At follow-up	6 (20)	29 (97)	4 (13)	5 (17)
	p value	0.73	<0.001	1.0	0.71
	Seroconversion	4 (13)	25 (83)	3 (10)	2 (7)
CVA16	Seropositivity				
	At enrollment	7 (23)	10 (33)	8 (27)	5 (17)
	At follow-up	9 (30)	11 (37)	30 (100)	7 (23)
	p value	0.77	1.0	<0.001	0.75
	Seroconversion	3 (10)	2 (7)	23 (77)	2 (7)
EV-A71	Seropositivity				
	At enrollment	5 (17)	17 (57)	6 (20)	18 (60)
	At follow-up	7 (23)	14 (47)	8 (27)	30 (100)
	p value	0.75	0.61	0.76	<0.001
	Seroconversion	4 (13)	1 (3)	3 (10)	17 (57)

*n = 30 for each virus. p values reflect the results of statistical analysis comparing the seropositive rates between the 2 time points (enrollment and follow-up) of the corresponding enterovirus serotypes. CV, coxsackievirus; EV, enterovirus.

At follow-up (>7 days after the onset of illness), seropositivity for homotypic viruses reached 97%–100% in all patient groups; comparisons for seropositivity rates at enrollment and follow-up were all significant (p<0.001) (Table 2), corresponding to seroconversion rates of 57% (17/30) for EV-A71, 77% (23/30) for CVA16, 83% (25/30) for CVA10, and 93% (28/30) for CVA6 (Table 2). We found no difference in antibody responses (seropositivity and seroconversion) between the groups of patients who were positive for EV-A71 subgenogroup B5 and those from whom EV-A71 subgenogroup was not available (data not shown).

**Figure 1:**
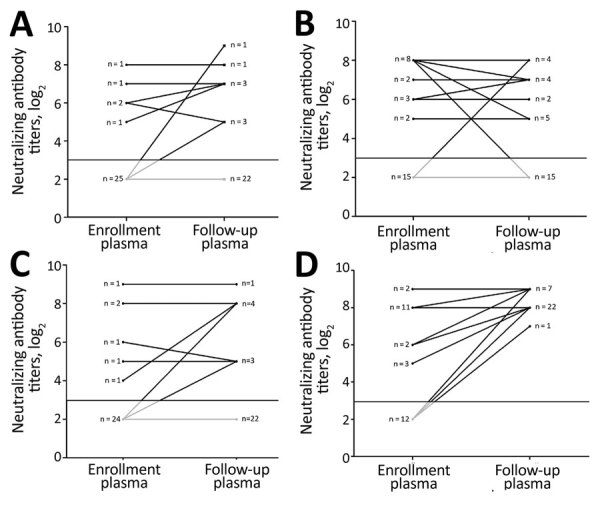
Kinetics of neutralizing antibody titers in plasma samples collected at enrollment and follow-up from patients infected with EV-A71 in study of patients with hand, foot and mouth disease, Vietnam. A) CVA6 (p = 0.073); B) CVA10 (p = 0.347); C) CVA16 (p = 0.250); D) EV-A71 (p<0.001). CV, coxsackievirus; EV, enterovirus.

**Figure 2:**
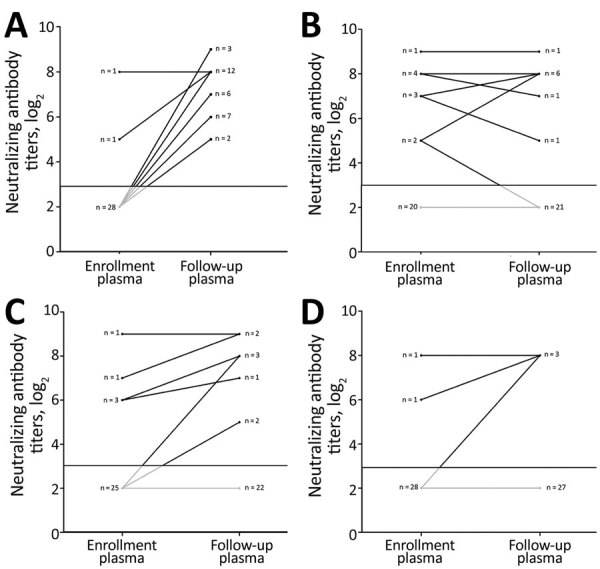
Kinetics of neutralizing antibody titers in plasma samples collected at enrollment and follow-up from patients infected with CVA6 in study of patients with hand, foot and mouth disease, Vietnam. A) CVA6 (p<0.001); B) CVA10 (p = 0.915); C) CVA16 (p = 0.021); D) EV-A71 (p = 0.5). CV, coxsackievirus; EV, enterovirus.

The difference in the proportion of patients who were seropositive for heterotypic viruses was not statistically significant between the enrollment and follow-up time points (Table 2). At follow-up, seroconversion for heterotypic viruses was recorded in only 3% (1/30) to 23% (7/30) of the patients, with comparable rates across serotypes (Table 2). For example, of the 30 patients infected with CVA16, seroconversions for CVA6 were recorded in 10%, seroconversions for CVA10 in 7%, and seroconversions for EV-A71 in 7%. Similarly, of the 30 patients infected with EV-A71, seroconversions for CVA6 were recorded in 13%, seroconversions for CVA10 in 3%, and seroconversions for CVA16 in 10%. Five patients became seronegative for heterotypic viruses at follow-up (Figures 1, 2; Appendix Figures 1, 2).

### Seropositivity versus Illness Day at Enrollment and Patient Age

**Figure 3:**
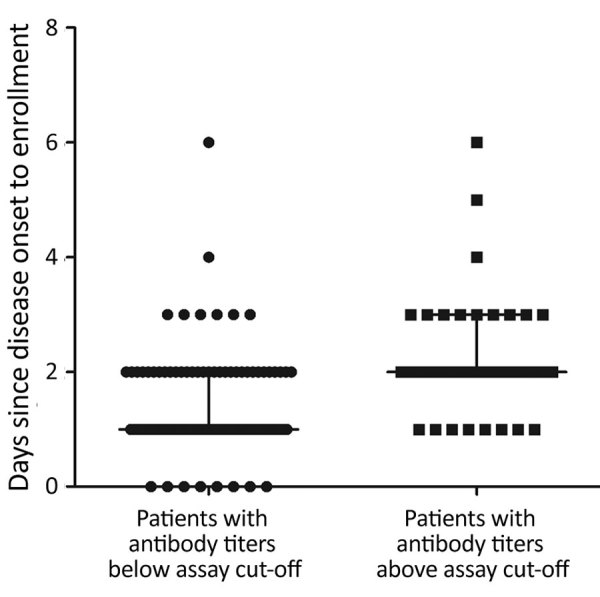
Association between antibody response (seropositive) and illness days at enrollment (p<0.001) in study of patients with hand, foot and mouth disease, Vietnam. There were 82 patients with antibody titers below assay cutoff, and 38 patients with antibody titers above assay cutoff.

We found a significant difference in illness days at enrollment between the groups of patients who were seropositive by neutralization testing for any homotypic virus and those who were negative, median day of illness 2 (range 1–6 days) versus 1 (0–6 days) (p<0.001) (Figure 3). Despite the small sample size, subgroup analysis demonstrated a similar association between illness day and seropositivity at enrollment among patients infected with EV-A71 and those infected with CVA6 (Appendix Figure 3). We found no difference in age (months) between the groups of patients with and without neutralizing antibodies at enrollment (data not shown).

### Kinetics of Neutralizing Antibody Titers

To further shed light on the kinetics of neutralizing antibody titers over the course of illness, we plotted and compared the neutralizing antibody titers against homotypic and heterotypic enterovirus serotypes at enrollment and follow-up time points (Table 3; Figures 1, 2; Appendix Figures 1, 2). Overall, the antibody titers to homotypic enteroviruses at follow-up were significantly higher than those measured at enrollment (p<0.001; Table 3). All patients at follow-up had antibody titers against homotypic viruses ranging from 32 to 512, well above the protective level as defined from vaccine trials (*12*–*14*) (Appendix Figures 1, 2).

**Table 3:** Geometric mean titers of neutralizing antibodies against enteroviruses in study of patients with hand, foot and mouth disease, Vietnam*

Virus	Samples	Geometric mean titer (95% CI) of neutralizing antibodies			
		CVA6	CVA10	CVA16	EV-A71
CVA6	Enrollment	4.9 (3.6–6.7)	13.3 (6.8–26.1)	6.9 (4.3–11.3)	5.0 (3.6–7.0)
	Follow-up	151.4 (113.0–202.8)	13.0 (6.4–26.2)	10.8 (5.6–20.8)	6.0 (3.8–9.7)
	p value	<0.001	0.83	0.021	0.50
CVA10	Enrollment	6.2 (3.9–9.7)	10.1 (5.7–17.7)	5.3 (3.8–7.2)	5.9 (3.8–9.7)
	Follow-up	8.7 (4.8–16.1)	245.5 (181.1–332.7)	6.3 (4.0–10.1)	7.6 (4.3–13.8)
	p value	0.063	<0.001	0.098	0.50
CVA16	Enrollment	9.0 (5.0–16.0)	13.6 (6.9–26.7)	10.5 (5.7–18.6)	7.4 (4.3–12.9)
	Follow-up	10.3 (5.7–18.7)	14.6 (7.5–28.5)	141.2 (104.547–191.0)	9.8 (5.1–18.7)
	p value	0.25	1.0	<0.001	0.098
EV-A71	Enrollment	6.6 (4.2–10.4)	23.2 (11.5–46.7)	7.6 (4.4–13.0)	37.7 (17.9–79.4)
	Follow-up	9.4 (5.3–16.7)	18.8 (10.0–35.4)	10.1 (5.4–18.7)	295.1 (260.6–334.2)
	p value	0.073	0.31	0.25	<0.001

*p values reflect the results of statistical analysis comparing the geometric mean titers between the 2 time points (enrollment and follow-up) of the corresponding enterovirus serotypes. CV, coxsackievirus; EV, enterovirus; HFMD, hand, foot and mouth disease.

Neutralizing antibody titers against heterogeneous serotypes measured at the 2 time points were not statistically different, with most patients (15/50–27/30 [50%–90%)]) having antibody titers below the assay cutoff (i.e., below the protective level). There was, however, a significant difference in antibody titer against CVA16 between the 2 time points among patients infected with CVA6 (p = 0.021; Table 3). Subanalysis did not show that samples positive for CVA6 were more likely to be positive for CVA16 or any other serotypes than samples that were negative for CVA6 (Appendix Table 1).

### Antigenic Difference between Subgenogroup B5 and Emerging Subgenogroup C4

**Figure 4:**
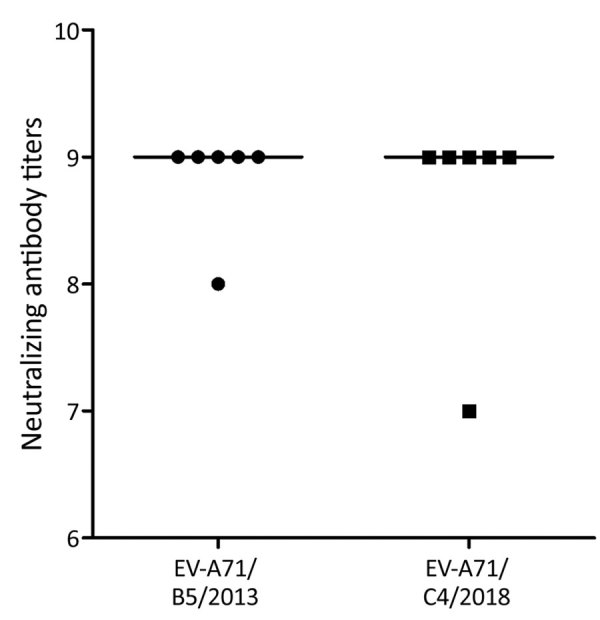
Neutralizing antibody titers (in binary logarithm) against subgenogroups C4 and B5 in follow-up plasma samples collected from 6 patients infected with EV-A71 subgenogroup B5 (p = 1.0) in study of patients with hand, foot and mouth disease, Vietnam. Information about years of collection is shown alongside the EV-A71 subgenogroup on the *x*-axis. EV, enterovirus.

To assess the extent to which infection with subgenogroup B5 circulating during 2013–2015 could elicit cross-neutralizing antibody responses against subgenogroup C4, which emerged in 2018 and caused a large outbreak of >130,000 hospitalizations and 17 deaths in Vietnam (*6*), we performed a complementary analysis using 6 follow-up plasma samples from the aforementioned group of patients infected with subgenogroup B5. Subsequently, all the included plasma samples could neutralize 2018 subgenogroup C4, and there was no difference in neutralizing antibody titers against the EV-A71 subgenogroups C4 and B5 (Figure 4). Likewise, the only available follow-up plasma sample collected from a patient infected with 2018 subgenogroup C4 had a neutralizing antibody titer of 1:512 against both subgenogroups.

## Discussion

Despite the public health threat of HFMD, scarce information exists for pathogen–pathogen and host–pathogen interactions, from the immunity perspective, to inform the development and implementation of intervention strategies, especially vaccines, and the design of epidemiologic research on disease surveillance and transmission dynamics. Here we report on the seropositivity, seroconversion, and neutralization in HFMD patients infected with EV-A71, CVA6, CVA10, or CVA16, the 4 most common enterovirus serotypes responsible for the ongoing HFMD epidemic in Vietnam and the Asia–Pacific region over the past few decades.

In terms of seropositivity, our results showed that antibody response against homotypic viruses at or above the titer of the protective level developed quickly after the onset of illness, with seropositivity for homotypic viruses changing from <60% at day 0–6 after illness onset (at enrollment) to 97%–100% at follow-up (7–19 days after the onset of illness). We could find no existing data obtained from natural infection to compare with our results. However, results obtained from phase 3 vaccine trials have shown that at day 56 after the administration of the first 2 doses of inactivated EV-A71 vaccine, 98.5–99.9% of the volunteers had neutralizing antibody against EV-A71 at titers of >1:16 (*12*–*14*). Collectively, these data expand our knowledge about immunogenicity elicited as a consequence of EV-A71 vaccination and natural infection. Coxsackievirus vaccine development has not gone beyond animal experiments; thus, no similar data exist for CVA6, CVA10, or CVA16 (*15*,*17*).

In contrast to the observed data for homotypic viruses, seropositive rates for heterotypic viruses were recorded in <57% of the patients during the course of illness. Furthermore, at follow-up, only a small proportion (3%–23%) of the patients had seroconverted for heterotypic viruses, suggesting that cross-neutralization among EV-A71, CVA6, CVA10, and CVA16 is absent or occurs in only a small proportion of patients (*24*,*25*). It cannot, however, be ruled out that these seropositive and seroconverstion rates, especially among CVA6 patients, were attributable to previous exposure or co-infection with other serotypes (e.g., CVA16 in the case of CVA6 patients), which may have been undetected by PCR. Our data support a recent report about recurrent HFMD episodes resulting from reinfection with heterotypic serotypes in China (*9*) and the absence of cross-neutralization among these 4 enterovirus serotypes observed in vaccine studies (*13*,*16*,*17*). As such, multivalent vaccines are needed to control HFMD.

EV-A71 exists as a single serotype but is genetically divided into several genogroups (e.g., A, B, and C) and subgenogroups (e.g., C1–C5 and B1–B5). In Vietnam, HFMD has been a major public health concern since 2011, causing an average of 80,000 hospitalizations per year. The switches between predominant EV-A71 subgenogroups have been well documented; C4 was responsible for the 2011–2012 outbreak (*18*) followed by the predominance of B5 during 2013–2015 (*10*) and the reemergence of C4 in 2018 (*6*). Of note, the emergence of C4 in 2018 resulted in a severe outbreak that caused >130,000 hospitalizations and 17 deaths. The underlying mechanism that determines the emergence of certain subgenogroups in specific localities remains a puzzle; it may be a consequence of a complex interplay among the pathogen, the hosts, and public health response, of which antigenic evolution might play a role (*26*,*27*). The fact that all serum samples from subgenogroup B5–infected patients collected before 2018 could neutralize the 2018 C4 virus suggests that immunity developed as a consequence of natural infection with subgenogroup B5 could provide protection against subgenogroup C4. However, the extent to which waning immunity (*28*), as observed from vaccine trials, may influence the long-term protection and overall population immunity, in turn resulting in possible reinfection, which has previously been reported in China (*9*), as well as disease emergence, remains unknown. The underlying mechanism determining the emergence of subgenogroup C4 in Vietnam in 2018 warrants further research.

Our study has some limitations. We based our analysis on only the 4 predominant serotypes currently responsible for the ongoing HFMD epidemics in the Asia–Pacific region, whereas >20 enterovirus serotypes have been reported to be associated with HFMD in the region. Furthermore, because of the unavailability of plasma samples, we were not able to informatively assess the antigenic relationship between EV-A71 subgenogroups responsible for major HFMD outbreaks in Vietnam since 2011 (C4 and B5) with proper sample size. Likewise, our syndromic hospital-based surveillance may have missed atypical HFMD cases. Together with the convenience sample used, these limitations may lower the level of generalizability of the obtained results to some extent. In addition, we were unable to obtain long-term follow-up blood samples from HFMD cases after hospital discharge. Therefore, evaluation of antibody kinetics and the waning antibody profiles of the natural infection beyond the sampling period of the current study remain unknown.

In summary, because human infection with 1 HFMD-causing enterovirus serotype can elicit neutralizing antibodies against homotypic viruses, our results support previous reports about the potential benefit of monovalent EV-A71 vaccine in reducing the incidence of EV-A71–associated HFMD (*29*). The data also emphasize the requirement for multivalent vaccines to control HFMD. Our results offer evidence that is essential for the development of intervention strategies, especially multivalent vaccines, and the design of seroepidemiologic studies.

Appendix Discussion of the microneutralization process in the study of enteroviruses in patients with hand, foot and mouth disease.
