# Supplementary material for: Neutralizing Antibodies against Enteroviruses in Patients with Hand, Foot and Mouth Disease
Source: Emerg Infect Dis. 2020 Feb;26(2):298–306. doi: 10.3201/eid2602.190721 (PMC6986819; doi:10.3201/eid2602.190721)
Supplement: Appendix — Discussion of the microneutralization process in the study of enteroviruses in patients with hand, foot and mouth disease. [file 19-0721-Techapp-s1.pdf]

Article DOI: https://doi.org/10.3201/eid2602.190721

Neutralizing Antibodies against Enteroviruses in Patients with Hand, Foot and Mouth Disease

Appendix

Microneutralization Procedure

1. For each tested serum, first dilute the serum 4× in maintained medium (MM), then inactivate the diluted serum sample at 56°C for 30 minutes.

2. Add 125 μL MM to all wells of a 96-well U-bottom plate. Additionally, as negative controls, add 125 μL of MM to wells H1–H12.

3. Add 125 μL of the 4× diluted serum prepared above to wells A1–A4. The serum sample is now diluted 8× (1:8) in quadruplicate.

4. Next, transfer 125 μL of the 1:8 diluted serum from wells A1–A4 to wells B1–B2. The serum sample is now diluted 16× (1:16) in quadruplicate.

5. Continue this 2× dilution (step 4) until wells G1–G4 (1:512); that is, transfer 125 μL of the 1:16 diluted serum to wells C1–C4, and so on. Then, pipet out 125 μL of the diluted serum in wells G1–G4.

6. Add 125 μL of the working dilution (100 $TCID_{50}$) of the virus stock to all wells of the 96-well U-bottom plate containing prepared diluted serum samples, except wells H1–H12, which serve as negative controls.

7. For back-titration, prepare 10-fold dilution series (10^{-1} to 10^{-4}) of the working dilution of the virus stock.

8. Incubate the plate containing diluted serum samples and virus prepared in step 6 and 10-fold dilution series of the working dilution of the virus stock (step 7) at 37°C for 1 hr.

9. Retrieve the 96-well U-bottom plates pre-coated with rhabdomyosarcoma (RD) cells at ≥90% of confluence (prepared a day in advance) from the incubator, and discard the growth medium.

10. Wash the cells 2 times with PBS (150 µL/well each time).

11. Transfer 50 µL of the serum–virus mixture and negative controls prepared in step 6, and 50 µL each virus dilutions prepared for back-titration to the corresponding wells of 96-well U-bottom plates containing the RD cells (step 10).

12. Incubate the plates at 37°C for 1 hr.

13. Refeed all wells with 130 µL of MM.

14. Incubate plates at 37°C and 5% CO_2 for 5 days.

15. Observe the cells daily for cytopathic effects and record the results.

Appendix Table 1. Assessment of cross-neutralizing effects among CVA6, CVA10, CVA16, and EV-A71.

	Sero-status against CV-A6		Sero-status against CV-A10		Sero-status against EV-A71	
Virus	positive, n (%)	negative, n (%)	positive, n (%)	negative, n (%)	positive, n (%)	negative, n (%)
CV-A16 positive serum samples, N = 50	24 (48)	26 (52)	24 (48)	26 (52)	20 (40)	30 (60)
CV-A16 negative serum samples, N = 70	29 (41)	41 (59)	29 (41)	41 (59)	25 (36)	45 (64)
P value	0.48		0.32		0.63	
OR (95%CI)	1.31 (0.63–2.71)		0.69 (0.33–1.44)		1.2 (0.57–2.54)	
	Sero-status against CV-A6		Sero-status against CV-A10		Sero-status against CV-A16	
	positive, n (%)	negative, n (%)	positive, n (%)	negative, n (%)	positive, n (%)	negative, n (%)
EV-A71 positive serum samples, N = 45	19 (42)	26 (58)	25 (56)	20 (44)	20 (44)	25 (56)
EV-A71 negative serum samples, N = 75	34 (45)	41 (55)	39 (52)	36 (48)	30 (40)	45 (60)
P value	0.74		0.71		0.63	
OR (95%CI)	0.88 (0.42–1.86)		1.15 (0.55–2.42)		1.20 (0.57–2.54)	
	Sero-status against CV-A6		Sero-status against CV-A16		Sero-status against EV-A71	
	positive, n (%)	negative, n (%)	positive, n (%)	negative, n (%)	positive, n (%)	negative, n (%)
CV-A10 positive serum samples, N = 64	27 (42)	37 (48)	24 (38)	40 (62)	25 (39)	39 (61)
CV-A10 negative serum samples, N = 56	26 (46)	30 (54)	26 (41)	30 (59)	20 (36)	36 (64)
P value	0.64		0.32		0.71	
OR (95%CI)	0.84 (0.41–1.73)		0.69 (0.33–1.44)		1.154 (0.55–2.42)	
	Sero-status against CV-A10		Sero-status against CV-A16		Sero-status against EV-A71	
	positive, n (%)	negative, n (%)	positive, n (%)	negative, n (%)	positive, n (%)	negative, n (%)
CV-A6 positive serum samples, N = 53	27 (51)	26 (49)	24 (45)	29 (55)	19 (36)	34 (64)
CV-A6 negative serum samples, N = 57	37 (55)	30 (45)	26 (39)	41 (61)	26 (39)	41 (61)
P value	0.64		0.48		0.74	
OR (95%CI)	0.84 (0.409–1.734)		1.31 (0.628–2.71)		0.88 (0.42–1.86)	

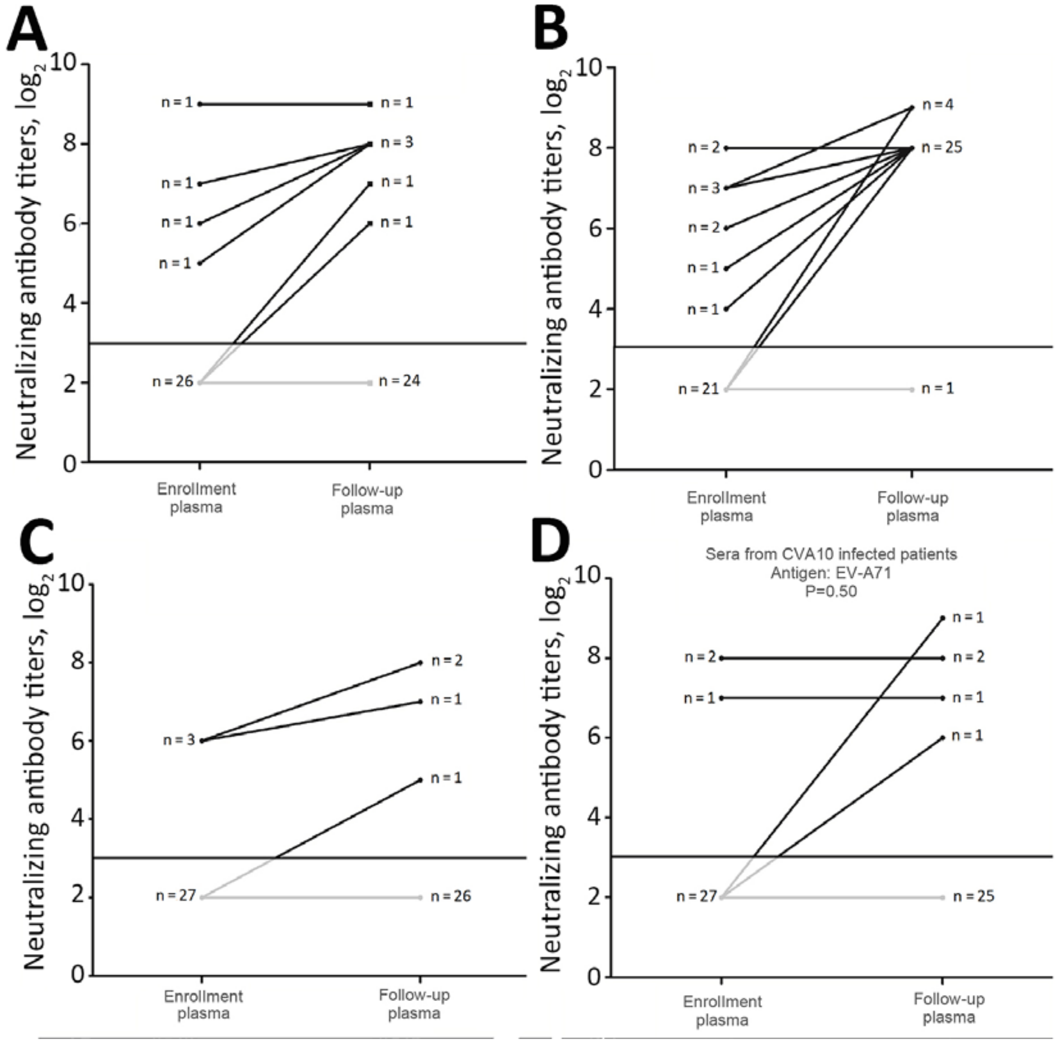

Appendix Figure 1. Graphs illustrating the kinetics of neutralizing antibody titers (in binary logarithm) against (A) CVA6 (p = 0.063), (B) CVA10 (p<0.01), (C) CVA16 (p = 0.098), and (D) EV-A71 (p = 0.50) in plasma samples collected at enrollment and follow-up from patients infected with CVA10.

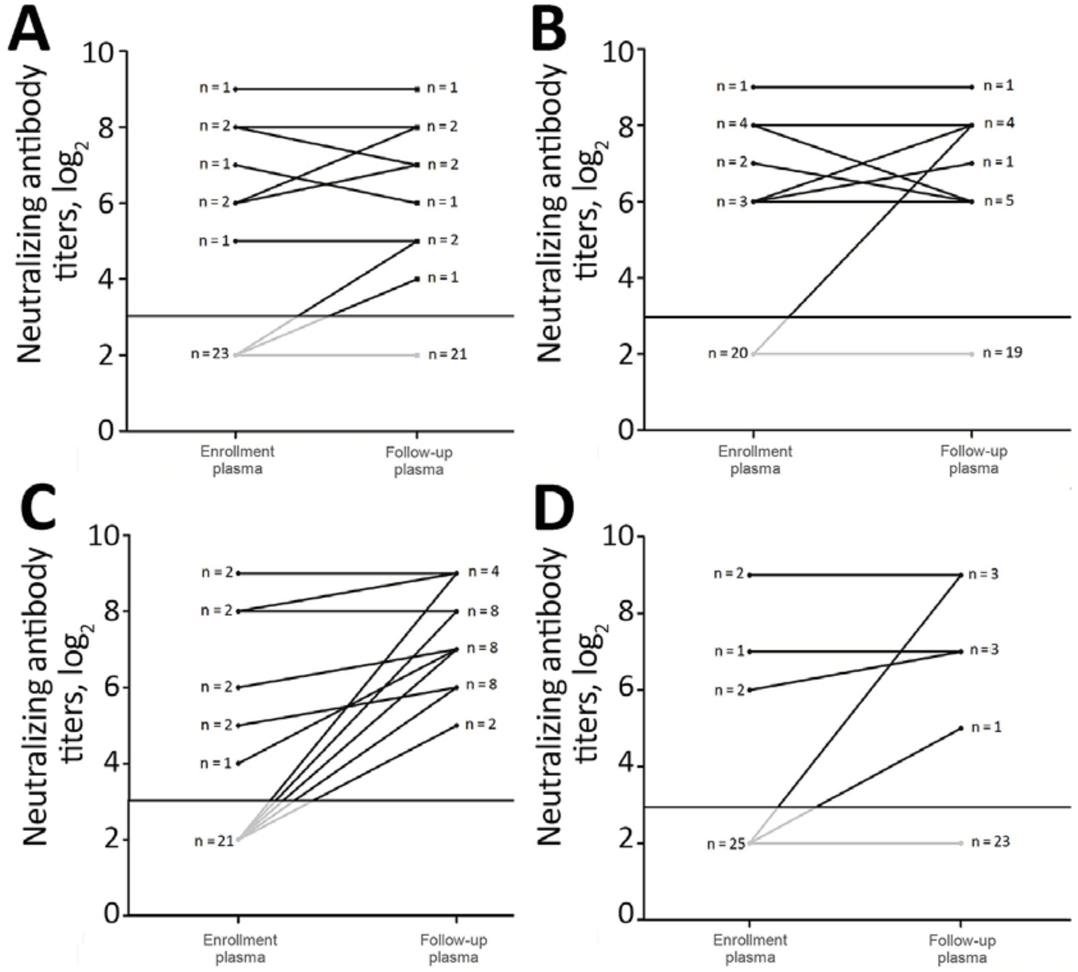

Appendix Figure 2. Graphs illustrating the kinetics of neutralizing antibody titers (in binary logarithm) against (A) CVA6 (p = 0.202), (B) CVA10 (p = 1.0), (C) CVA16 (p<0.001), and (D) EV-A71 (p = 0.098) in plasma samples collected at enrollment and follow-up from patients infected with CVA16.

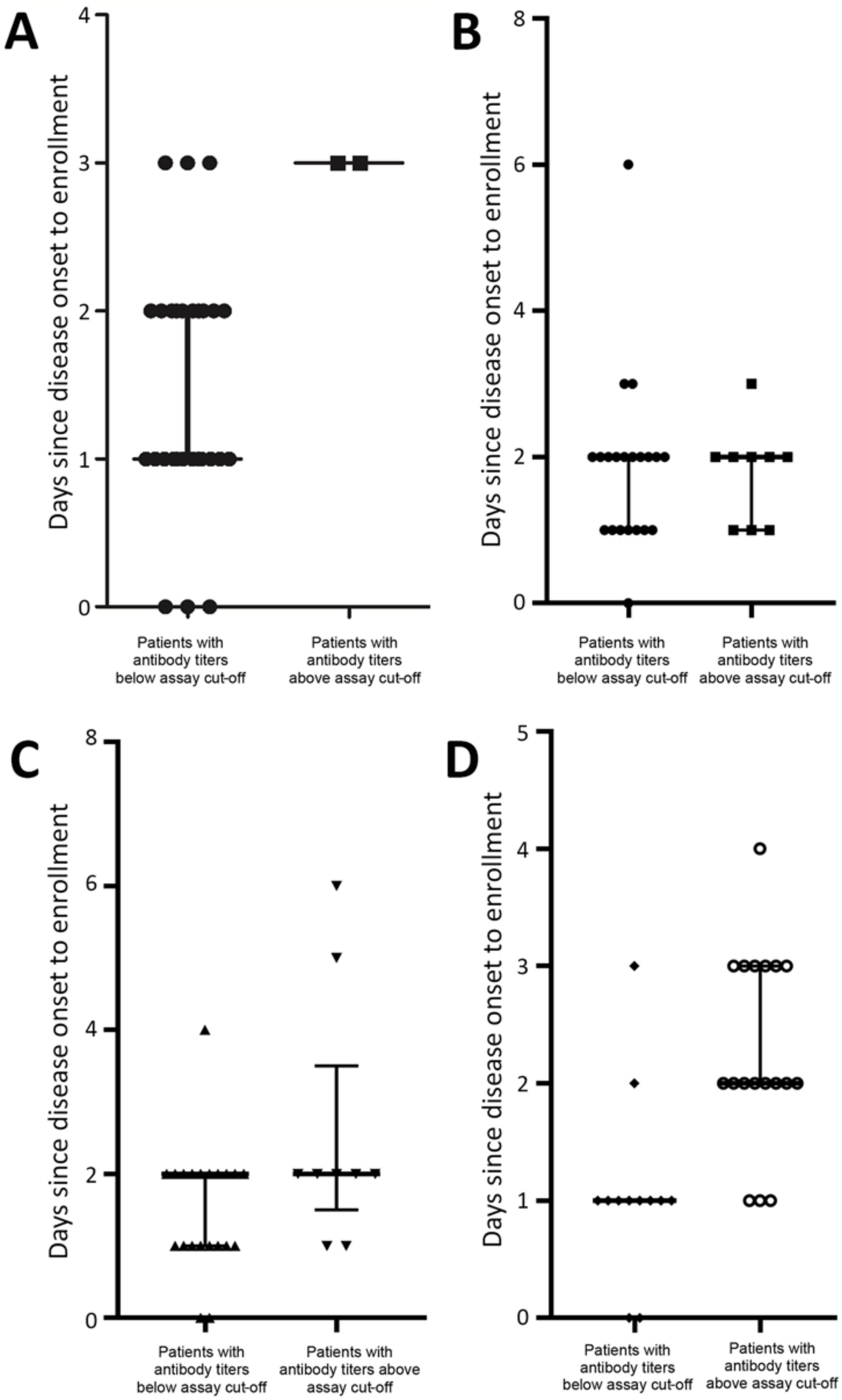

Appendix Figure 3. The association between antibody response (seropositive) and illness days at enrollment for individual groups of patients infected with (A) CVA6 (p = 0.028), (B) CVA10 (0.894), (C) CVA16 (p = 0.125), and (D) EV-A71 (p = 0.001).
